# Ionic Liquids and Deep Eutectic Solvents for CO_2_ Conversion Technologies—A Review

**DOI:** 10.3390/ma14164519

**Published:** 2021-08-11

**Authors:** Kranthi Kumar Maniam, Shiladitya Paul

**Affiliations:** 1 Materials Innovation Centre, School of Engineering, University of Leicester, Leicester LE1 7RH, UK; km508@leicester.ac.uk; 2 Materials and Structural and Integrity Technology Group, TWI, Cambridge CB21 6AL, UK

**Keywords:** renewable energy, electrochemical conversion, carbon dioxide reduction, functionalized ionic liquids (ILs), carbon dioxide transformation

## Abstract

Ionic liquids (ILs) have a wide range of potential uses in renewable energy, including CO_2_ capture and electrochemical conversion. With the goal of providing a critical overview of the progression, new challenges, and prospects of ILs for evolving green renewable energy processes, this review emphasizes the significance of ILs as electrolytes and reaction media in two primary areas of interest: CO_2_ electroreduction and organic molecule electrosynthesis via CO_2_ transformation. Herein, we briefly summarize the most recent advances in the field, as well as approaches based on the electrochemical conversion of CO_2_ to industrially important compounds employing ILs as an electrolyte and/or reaction media. In addition, the review also discusses the advances made possible by deep eutectic solvents (DESs) in CO_2_ electroreduction to CO. Finally, the critical techno-commercial issues connected with employing ILs and DESs as an electrolyte or ILs as reaction media are reviewed, along with a future perspective on the path to rapid industrialization.

## 1. Introduction

Controlling greenhouse gases (e.g., CO_2_), which are often associated with energy generation and consumption, is the most challenging environmental issue and a source of great concern around the world [1,2]. Sustainable energy derived from renewable sources is an appealing option for mitigating global climate change. Significant efforts have been made to minimise reliance on fossil fuels by developing renewable energy sources but the contribution from these sources are in the range 25–28%, a 3% increase since 2019 [3,4]. As a renewable carbon source, converting or transforming CO_2_ into an energy carrier as a fuel, fuel additive, or value-added chemical using renewable electricity could contribute to mitigating climate change and attaining a carbon-neutral economy [5,6]. Ionic liquids (ILs) are a new class of compounds that, due to their tunable physicochemical properties, have the potential to be used as novel materials in renewable energy applications such as CO_2_ conversion [7,8,9,10].

During conversion, CO_2_ is utilized as a feedstock to produce a wide range of fuels, fuel additives, acids, alcohols through formation of different chemical bonds (C–C, C–H, C–N, C–O). Direct reduction utilising homogeneous or heterogeneous catalysts converts CO_2_ to CO and small organic molecules. Further, CO_2_ is also converted to organic compounds such as carbonates, carboxylates, and carbamates by participating in an electrosynthesis process with an organic substrate (such as ethylene oxide, propylene oxide, olefins, and amines). Such synthesis is usually carried out in the presence of an alkylating agent, termed as CO_2_ electro-organic transformation (referred as “transformation” in the review). Besides, storage of thermal energy in solar is one field where ILs due to their high temperature thermal stability are able to store a considerable amount of heat. Numerous ILs can theoretically be synthesised by combining different cations and anions, providing a good platform for design [11].

**Figure 1:**
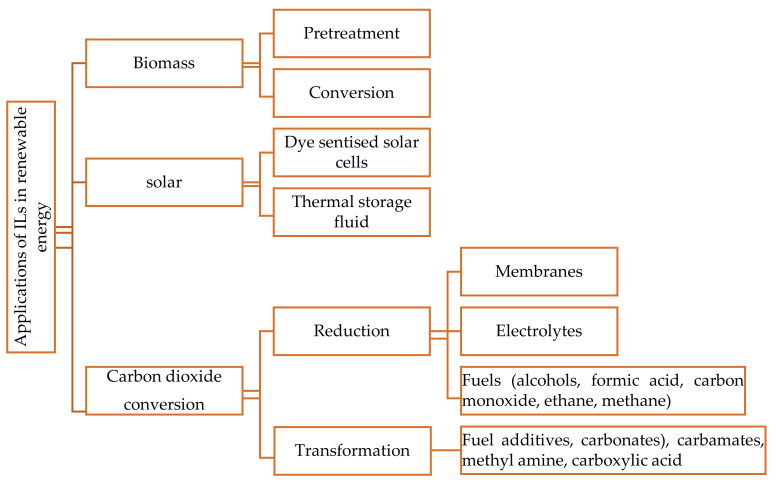
Applications of ionic liquids in renewable energy.

The performance of ILs can be credited to their ability to improve solubility, activation, and electrochemical conversion of CO_2_ under moderate reaction conditions to fuels. This makes ILs appealing as an alternative media. Furthermore, typical electrolytes used for CO_2_ conversion have drawbacks such as high volatility, separation behaviour, corrosivity to metals, and instability in electroreduction or transformation, which motivated the scientific community to focus on ILs [7,10,11,12,13,14]. There have recently been numerous studies on the utilisation of CO_2_-saturated ILs as electrolytes to enhance the electroreduction of CO_2_ and electrosynthesis of organic chemicals (such as carbonates, carbamates, etc). Recently, ILs have been explored as thermal energy storage fluids, and are becoming a promising research topic [11]. Because of their high ionic conductivities and wide electrochemical windows, as well as their increased solubility relative to traditional solvents, ILs can play a critical role in CO_2_ conversion. Figure 1 depicts the use of ILs in renewable energy applications.

This article focuses on improvements in the role of ILs, DESs in renewable resources, such as CO_2_ reduction and transformation to value added chemicals. The primary goal is to provide a critical review of the new challenges and prospects of ILs for adopting sustainable processes of renewable energy. CO_2_ utilization via electrochemical route has been, and is a hot topic, with ILs, gaining increased attention. There are a lot of reviews published in the electrochemical reduction of CO_2_ using ILs [10,15] and the electrosynthesis of organic compounds using CO_2_ [8,9]. However, reviews on the combination of CO_2_ reduction and transformation employing ILs and deep eutectic solvents (DESs) are very scarce. The current review presents a synopsis of the considerable progress achieved by ILs in renewable energy applications, with a focus on CO_2_ conversion and transformation. In addition, the new developments associated with deep eutectic solvents (DESs) are also covered in this review. Unlike the other reviews in the field, we have taken a different approach in consolidating the results from the fields proposed. Additional information on the technical challenges, cost considerations that are expected to be solved, which were not sufficiently covered in previous reviews and works, will be presented. The ionic liquids considered in this review are listed in Table 1.

## 2. Carbon Dioxide Conversion in ILs

### 2.1. Conversion by Electroreduction

Although the use of CO_2_ as a raw material appears to be particularly promising, CO_2_’s inert nature, along with its high thermodynamic stability, poses a challenge to CO_2_ conversion, transformation, and utilisation as an effective renewable energy source. Numerous CO_2_ reduction strategies, such as thermal, biochemical, photochemical, and electrochemical approaches, have been studied extensively, with various degrees of success and practicality [23]. Among them, electrochemical CO_2_ conversion, either by electroreduction or electro-transformation, to value-added chemicals and fuels has sparked great interest for sustainable energy conversion and storage [24]. The key advantages with the electrochemical conversion of CO_2_ is the tunability of the reactions by adjusting the electrolytes, operating conditions and electrode materials.

**Figure 2:**
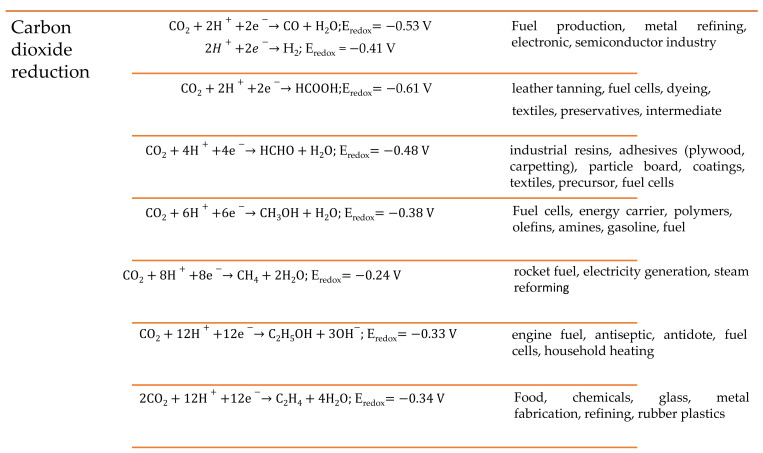
Summary of the possible CO_2_ reductions with their redox potentials and applications.

As an important component in the electroreduction process, the electrolyte interacts with the electrode surface, reactants, and intermediates, which plays a critical role in charge transport [25]. Depending on the electrochemical reduction routes involved in the process, changes in CO_2_ solubility, conductivity, and viscosity are thought to have a substantial impact on the catalytic activity for electroreduction and electro-transformation into alcohols, alkanes, alkenes, acetates, formates, and organic carbonates (cyclic, dialkyl). Figure 2 depicts the possible formation of CO_2_ electroreduction products and their accompanying redox potentials (vs. SHE at pH = 7) along with the applications of the relevant products. While the primary role of an electrolyte is to conduct the ionic charge between the electrodes, it needs to satisfy certain criteria such as (i) solubility for CO_2_, (ii) compatibility with electrode materials (especially cathodes), (iii) stability (without decomposition), (iv) safety in handling and storage. Achieving these criteria will not only make them function as a good electrolyte but also improve the overall efficiency of the process. Because of their high ionic conductance, aqueous electrolytes containing inorganic salts are the most often employed electrolytes. However, they have poor CO_2_ solubility (0.03 mol L^−1^ CO_2_ in water under 298 K, 0.1 MPa), low conversion rates (30 percent at 1 A cm^−2^) with substantial hydrogen evolution as a side reaction, and an unsatisfactory applicable potential range [26].

#### 2.1.1. CO_2_ Electroreduction in Ionic Liquids

To overcome the limitations of aqueous electrolytes, such as poor CO_2_ solubility, hydrogen evolution at the cathode, and low conversion efficiency, organic solvents such as acetonitrile, dimethyl sulfoxide, polycarbonates, and dimethylformide were utilised as non-aqueous systems to improve CO_2_ conversion. These have been shown to offer better CO_2_ solubility than aqueous electrolytes while also having good recyclability. However, significant shortcomings of organic solvent electrolytes, such as their high volatility, and poor safety characteristics (flammability, toxicity), have limited their commercialization and applicability. Furthermore, the recycling costs of organic solvents employed in electrolyzers remain high due to their potential miscibility with the target products [27]. This motivated the scientific community to pursue research in ionic liquids as alternate potential electrolytes to organic solvents, aqueous media (containing inorganic salts). The key benefits are their tunable features, such as polarity, hydrophobicity, and solvent miscibility, which can be achieved by modifying the arrangement of the cations and anions [7]. Ionic liquids have recently attracted a lot of interest as electrolytes because of their high CO_2_ adsorption capacity, solubility, selectivity to CO_2_ over other gases (such as N_2_, O_2_, CH_4_), and low energy consumption. They are a very diverse and efficient family of promoters in the electrochemical reduction of CO_2_, with the ability to improve reaction characteristics by changing their interaction and exhibiting high electrochemically stable windows (4–5 V), high ionic conductivities, and low vapour pressures [10,15]. Also, it has been observed that ILs can lower the energy barrier of reactions by building complexes with the intermediates generated during the reaction [28].

Zhao et al. [29] employed a [BMIm][PF_6_] electrolyte to make syngas (CO + H_2_), a value-added fuel, and demonstrated the promise of an ionic liquid for CO_2_ conversion applications. This was claimed to be one of the earliest works that employed ionic liquid as an electrolyte. Besides, the study confirmed the synthesis of small organic compounds such as formic acid in lower concentrations. Rosen et al. used an IL-mediated selective conversion strategy to reduce the high overpotential that is commonly observed with CO_2_ reduction to CO in aqueous systems, where the IL was demonstrated to lower the energy required to generate the (CO_2_•^−^) intermediate [28]. When [EMIm][BF_4_] was introduced as an electrolyte to the reaction system, electrochemical characterization results showed that the overpotential could be reduced by up to 0.2 volts with silver serving as the cathode. The authors attributed the reduction in overpotential associated with the electroreduction of CO_2_ to CO to the complexation between CO_2_ and [BF_4_]^−^. This complex was shown to play a critical role in lowering the energy that is required to break the chemical bonds in CO_2_ to form the (CO_2_•^−^) intermediate, and achieved a continuous production of CO up to 7 hours with a Faradaic efficiency of ~96% [30]. Since then attempts were made with imidazolium cations with different anions [BF_4_]^−^, [CH_3_COO]^−^, [Tf_2_N]^−^, [PF_6_]^−^, [TfO]^−^ using different catalyst systems such as 2D dichalcogenide structures (MoS_2_ [31], WS_2_ [32]), doped carbons [33], metals, metal-alloys. One of the significant works was reported by Sun et al. [19] which focused on imidazolium based ILs with different anions: [BF_4_]^−^, [PF_6_]^−^, [TfO]^−^, [Tf_2_N]^−^,and [DCA]^−^ with [BMIm]^+^ as the common cation. N-doped carbon on carbon paper which can exhibit the feature of a graphene was used the catalyst. The results demonstrated a conversion of CO_2_ into CH_4_ with fluorine-based ones displaying higher total current densities than the non-fluorine ones. The authors explained this to the strong interactions between CO_2_ and fluorine, which weakens the C=O bond by forming a Lewis acid-base adduct and the fact was also supported by other reference works. In addition to the typical ILs with “common” anions, Snuffin et al. developed and synthesized a novel imidazolium based IL with dual halide anion combination: 1–ethyl–3–methyl–imidazolium trifluorochloroborate [EMIm][BF_3_Cl], demonstrated a strong CO_2_ solubility and also a positive reduction potential of −1.8 V while promoting electroreduction of CO_2_ [34].

Although there are many advantages in using ILs as electrolytes in electroreduction of CO_2_ besides excellent physico-chemical properties such as high reactant solubility, lowering the energy barrier, the relatively high cost and their associated viscosity of ILs hinder their practical application. Table 2 provides a summary of some ILs reported as electrolytes for electroreduction of CO_2_ with different catalyst combinations. As can be seen from the Table 2, it is clear that imidazolium-based ILs have been the most investigated type of IL. Especially, [EMIm][BF_4_], followed by [BMIm][BF_4_] and [BMIm][PF_6_], have been, by far, the most widely used ILs in the electroreduction of CO_2_. [BF_4_]^−^, [PF_6_]^−^, [TfO]^−^, and [Tf_2_N]^−^ are the most commonly used anions as can be seen from the table and reported by many works. This can be related to the Lewis acid-base interaction effect between the selected anion X^−^ (X: [BF_4_]^−^, [PF_6_]^−^, [TfO]^−^, [Tf_2_N]^−^) and CO_2_ molecule forming [X^—^CO_2_]^−^ complex. Such a complex displays strong alkalinity and tends to displace the bonds that exist between the inert anions (B−F, P−F, C−F, and S=O). Also, these anions possess weak ionization characteristics and weak coordination capacity and as a result promote the electrochemical reduction of CO_2_ by favouring the interactions between CO_2_ and metal electrode without affecting the reaction characteristics. Amongst the anions, imidazolium cations with [BF_4_]^−^ anions were reported high Faradaic efficiencies of >98% owing to their strongest Lewis acid-base interactions.

The Tanner group [18] investigated the effect of several cations: [EMIm]^+^, [BMIm]^+^, [PMIm]^+^, [BMPyl]^+^ on the performance of electrochemical CO_2_ reduction using silver electrodes as the catalyst. Since comparison, analysis of the data in terms of potentials could not demonstrate the influence of imidazolium cations towards the performance, studies were extended with different anions: [BF_4_]^−^, [Tf_2_N]^−^, [FAP]^−^ with [BMIm]^+^ as the cation. [BMIm][FAP] based IL displayed the best reactant solubility amongst the others but with a lower current density. Bruzon et al. [43] investigated CO_2_ electroreduction in nitrogen-based imidazolium-based ILs with [FAP]^−^ as the anion, observed a significant reduction in the electric potential which is subsequently utilized to reduce CO_2_. It has been demonstrated that the functional groups: –OCH_3_, –CN minimised the free energy to form the first intermediate of CO_2_ reduction, (CO_2_•^−^) to a greater extent. Since the mechanism is not clearly understood, it is widely assumed that the structure of the IL might have more influence on the CO_2_ reaction than the reactant solubility.

#### 2.1.2. CO_2_ Electroreduction in Deep Eutectic Solvents (DESs)

Deep eutectic solvents (DESs) are obtained by combining a hydrogen bond acceptor and donor in specific mole ratios. These mixtures exhibit low melting points, similar properties and characteristics to ILs that are required for the electrochemical reduction of CO_2_ [44]. Also, these are less expensive and considered to be potential alternatives to ILs. Verma et al. [45] conducted experiments employing [EMIm]-based ILs and choline chloride:urea (ChCl:Urea—1:2) DESs as the electrolyte media for the electrochemical reduction of CO_2_. The results showed low conductivity and performance which increased on adding potassium chloride (KCl) to the non-aqueous electrolyte. Vasilyev et al. [17] studied the electrochemical reduction of CO_2_ employing different ChCl-based DESs, imidazolium chloride based DESs. Imidazolium chloride-based DESs were prepared by mixing IL chloride with ethylene glycol, polyethylene glycol-200 (PEG-200) as hydrogen bond donors. The results demonstrated that choline-based DESs, IL-chlorides with EG facilitated the electrochemical reduction of CO_2_, when silver is used as the catalyst. However, certain mixtures were shown to be non-room temperature liquids, which on addition of organic solvents could facilitate the electrochemical reduction of carbon dioxide with improved reactant (CO_2_) solubilities. Hydrogen evolution reaction was observed by Vasilyev et al. [17], Verma et al. [45] when water up to 15 vol % was added to ChCl-based DESs while displaying high FE with CO as the major product. The presence of the hydroxy group in the structure of the imidazolium cations, choline based DESs, was shown to be the key factor in enhancing the electrochemical reduction of CO_2_.

In recent times, the modification or preparation of catalytic electrode materials using ILs/DESs have gained primary attention as they are expected to reduce the background current of the electrode, optimize the performance of the electrode materials and favour the catalytic reduction of CO_2_. Besides, ILs/DESs can also be used as a medium to prepare catalysts. Bohlen et al. [46] performed the electrodeposition of indium from 1:2 M choline based DES (ChCl:EG—1:2), employed them as an electrocatalyst for the electrochemical reduction of CO_2_ to formate. As per the Cui et al. [15] review, this was the first publication which reported on the preparation of CO_2_ reduction catalysts by electrodeposition in DESs. Extending this method by tailoring the DES electrolyte properties and the electrodeposition conditions, it is possible to develop other metals with different sizes, shapes and structures and faces.

**Figure 3:**
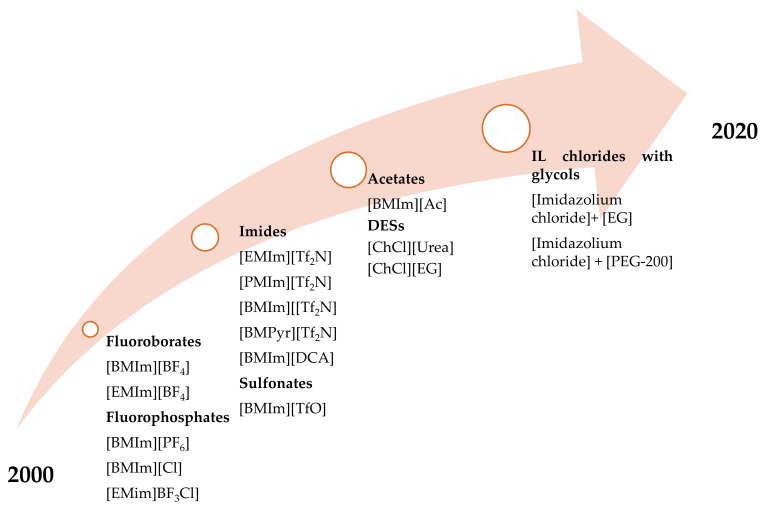
Developments of the ILs, DESs in CO_2_ electroreduction covering the period 2000–2020.

This provides a new research idea to produce selective products such as ethylene for future exploration in this field considering the combined advantages with ILs/DESs and electrodeposition. Table 3 lists few of the works studied using choline-based DESs and imidazolium chloride-based ones with different hydrogen bond donors. One common feature amongst all the studied DESs is the reactor type and the major product associated with the CO_2_ electroreduction. Figure 3 highlights the developments of the ILs, DESs that are employed for the electroreduction of CO_2_ using different classes of catalysts.

### 2.2. Conversion by Electrotransformation

**Figure 4:**
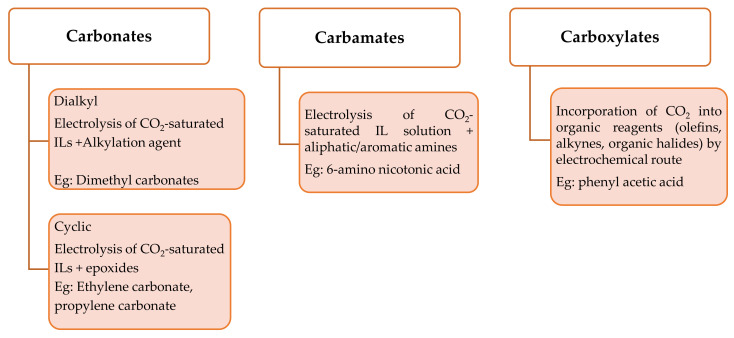
Summary of the organic products that were obtained from electrosynthesis via the CO_2_ transformation route [50].

An another effective method of utilising CO_2_ is to electrosynthesize the C_1_ feedstock into valuable fuel additives without the use of a hydrogen source [9,49]. In general, this non-hydrogenation process with mild working conditions converts CO_2_ into a diverse array of organic compounds such as carbonates (cyclic, dialkyl), carboxylic acids, and carbamates. Carbonates, specifically cyclic and dialkyl ones were identified to be the most effective fuel additives. It is possible to electrosynthesise numerous kinds of products via the transformation pathway through electrochemical processes involving CO_2_. A wide variety of substrates such as epoxides, alcohols, amines, aryl halides, and olefins have been used to transform the CO_2_ into their respective organic compounds [9,50,51,52]. This approach is thought to be the most efficient for some reactions that are thermodynamically unfavourable in the absence of external energy and when thermal catalysis options are severely constrained. The use of an electrochemical approach to synthesise organic compounds has several merits, including moderate conditions, high functional group tolerance, and inherent scalability and sustainability [8]. The general pathway of electrosynthesis of products (such as carbonates, carbamates, and carboxylates) via CO_2_ transformation involves the generation of electro-induced radical/anion from CO_2_-saturated with ILs and/or substrates. Subsequently, the generated radical/anion reacts with other substrates, yielding either of the transformed products mentioned above. Several investigations on the studies performed in this field have further shown that ILs have a stabilization effect on the electro-induced CO_2_ molecule or substrates (epoxides, ketones, alkenes, etc) radical/anion. When combined with the high reactant CO_2_ solubility and favourable electrochemical properties required for CO_2_ transformation, ILs are viewed as an alternative eco-friendly and prospective reaction medium to the currently utilized hazardous volatile organic solvents [51]. Previous review papers [8,9,50,52,53] address the significant research published in this domain, with a focus on the electrosynthesis of carbonates via the transformation route. As a result, this section presents a succinct overview of the most recent developments in the value-added chemical compounds generated by electrosynthesis via the CO_2_ transformation route in ILs other than carbonates. Figure 4 depicts the product classes that are generally produced from electrosynthesis via the transformation pathway utilising CO_2_-saturated with ILs.

Carbonates produced from electrosysnthesis reactions are obtained by the transformation of CO_2_ with organic substrates (epoxides, olefins). These provide a green synthesis pathway via the C^—^O bond formation which avoid the use of phosgene or CO, that are generally toxic in nature. These can be cyclic or dialkyl and have gained widespread interest in applications such as battery electrolytes, intermediates, polar aprotic solvents, and so on. Typical examples of cyclic carbonates include propylene carbonate, methylene carbonate while dimethyl carbonate stands out to be a classic example of a diakyl carbonate. Yuan group performed the electrosynthesis of cyclic carbonates utilising ILs saturated with CO_2_ and epoxide [54]. Under mild operating conditions, the reaction was studied using an undivided cell with a Cu working electrode and an Al or Mg rod sacrificial anode. The performance of CO_2_ cycloaddition to various epoxide substrates (propylene oxide, epichlorohydrin, and styrene oxide) was evaluated.

The best results were shown to be obtained by using propylene oxide as the substrate and [BMIm][BF_4_] as the reaction media, which resulted in 92% conversion and 100% selectivity to the desired product (cyclopropylene carbonate). CO_2_ underwent a one-electron reduction to generate the (CO_2_•^−^) radical anion, which subsequently interacted with the activated substrate to produce the matching cyclic carbonate. Wang et al. reported the electrosynthesis of butylene carbonate (cyclic carbonate) from diols (1,2-butanediol) in CO_2_-saturated imidazolium based IL ([BMIm][BF_4_]) in an undivided cell under mild operating conditions (1 atm, 50 °C). However, the highest yield that could be achieved employing the proposed IL system was 12%, with magnesium anode and copper as the cathode [55]. Under mild conditions, at atmospheric pressure and temperatures of 55 °C, Zhang et al. [56] developed a novel electrochemical technique to synthesise dialkyl carbonates from a CO_2_-saturated imidazolium based IL:[BMIm][BF_4_] in the presence of an alkylating agent. The corresponding process eliminates the use of organic solvents and supporting electrolytes. Most of the literature demonstrated that the CO_2_ transformation in IL was shown to be relatively easier than that in organic solvents. This was attributed to the formation of a ( [BMIm]^+^–[CO_2_^−^]) ion-pair [56], which could facilitate the better yield of the carbonates from electrosynthesis.

Dimethyl carbonates are considered to be an important class of dialkyl carbonates which serve as methylation agent and replace toxic substances such as phosgene, tertiary butyl methyl ether, methyl halides. The Wu group [55] demonstrated the utilization of N-heterocyclic carbenes (NHC), electrogenerated from CO_2_-saturated [BMIm][X]—ILs ([X] = [BF_4_]^−^, [PF_6_]^−^), and alcohols for the synthesis of dialkyl carbonates under mild conditions. During the process, the IL served as both a green solvent with low toxicity and also generated NHC. This eliminated the use of toxic organic solvents, and the addition of supporting electrolytes while favoring good conversion, selectivity. Since then there were numerous works published in this area which focused on the transformation of CO_2_ via the electrosynthesis route to the cyclic carbonates such as propylene carbonate using Lewis acid ILs, ILs with hydrogen bond donors. The high viscosities of ILs, combined with their low CO_2_ conversion, limit their direct application. This has motivated researchers to extend the studies to binary IL systems using either water or an organic solvent as a co-solvent. Functionalizing the ILs or changing the IL network with –OH or –COOH via hydrogen bonding was demonstrated to have a greater influence on the electrosynthesis process than unmodified ILs. Carbamates, carboxylates and anilines have significant attention as a value-added chemical for applications ranging from pharmaceuticals, agro chemicals, dyes, perfumes, intermediates for detergents and so on. Producing them via the electrosynthesis can be a beneficial way in terms of environment friendliness, moderate reaction conditions, eliminating the use of a toxic compound phosgene. To produce these compounds, it is necessary to transform the CO_2_ and its corresponding substrate, create either a C–N bond using amines (for carbamates) or C–C (for carboxylates). Table 4 lists an overview of the various organic compounds produced by electrosynthesis in CO_2_-saturated ILs along with the substrates, reactor types, catalysts employed in the process.

Carbonates (cyclic/dialkyl) are produced when CO_2_ is coupled to an alcohol during electrosynthesis, while coupling to an amine (aliphatic/aromatic) yields carbamates. Electrocarboxylation involves the coupling of CO_2_ to a radical/anion produced during the electrochemical reduction of organic halides (alkyl/aryl), thereby yielding carboxylate derivative. Feng’s group [66] performed the electrolysis at 50 °C in an undivided cell with Pt cathode and Mg anode at certain concentrations of acetophenone-an aromatic ketone, in [BMIm][BF_4_] saturated with CO_2_. This was carried out in the presence of the alkylating agent, methyl iodide (CH_3_I) to afford the corresponding α-hydroxycarboxylic acid methyl ester with yields of ~56–62%. The corresponding alcohols were obtained as the main by-products. Zhao et al. [20] investigated the effect of proton availability in ILs on the product distribution of acetophenone during electrocarboxylation with CO_2_. They observed that dry pyrolidinum-based IL [BMPyl][Tf_2_N] with limited proton availability was an appropriate medium for their electrocarboxylation system, yielding 98% 2–hydroxy–2–phenylpropionic acid. The competing reactions are not beneficial to the electrocarboxylation and some studies suggested that the product distribution is strongly dependant on the medium. Lu et al [58] studied the formation of phenylacetic acid in CO_2_-saturated [BMIm][BF_4_] via electrocarboxylation of benzyl chloride with silver cathode as the catalyst. Initially, benzyl chloride was electroreduced to its corresponding radical and subsequently coupled to CO_2_ to yield the carboxylate derivatives. Hiejima et al. [59] reported the synthesis of α-chloroethylbenzene via electrocarboxylation in [DEME][Tf_2_N]-based ionic liquid compressed with CO_2_. The experiments were carried out using a Pt cathode and a Mg anode at various temperatures and pressures; but obtained poor carboxylic acid product yields (~20%). Atobe et al [67] improved the yield further for the same process to 50% by using supercritical CO_2_ in [DEME][Tf_2_N]-based IL. However, obtaining the products via the transformation approach is a greener and easier option because it:

- avoids the use of hazardous reagents such as phosgene and cyanide
- simplifies the purification of the resultant products utilising single step chromatographic separations.

## 3. Techno-Commercial Challenges and Future Road Map

**Figure 5:**
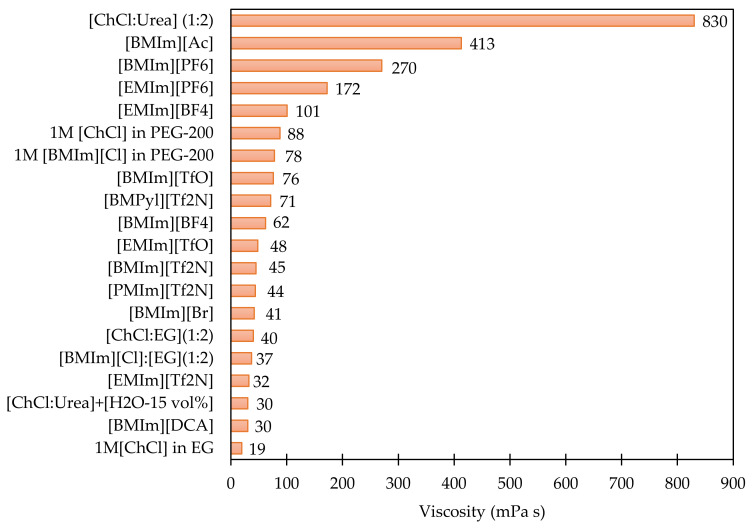
Plot comparing the viscosity of the ILs, DESs that are used in electrochemical conversion of CO_2_ at 298 K.

A large array of ILs in the range of ~10^18^ were discovered through different arrangements of cations and anions, which can possibly be synthetized and present a good platform for design [68,69,70], but, it is interesting to note that only ~10^2^–10^3^ ILs were commercialised. This could be ascribed to the complexities in the methods of preparation, expensive equipment, controlled operating conditions, availability of raw materials, costs, storage and safe handling after the synthesis, and limited availability of the physical, chemical property data base [71,72,73,74,75,76]. Most of the properties available so far are derived using the machine learning methods [77]. In addition, studies and data concerning the environmental considerations, recycling, biodegradability, recovery and reuse, thermal stability is very limited and lacks significant consideration [78]. Also, the high viscosities of traditional ILs limit their direct application for the electrosynthesis of organic molecules from CO_2_, electroreduction of CO_2_ to CO and other chemicals, which is the main reason why binary IL systems have received a lot of significant interest recently. The higher viscosity of ILs pose the difficulties in transportation of the reactant species (CO_2_) despite their high solubility. As a result, from the standpoint of functioning in a real-world application, a simple and efficient system is still required. In the recent times, DESs prepared by mixing imidazolium chloride ILs with hydrogen bond donors such as [ChCl:Urea], [ChCl:EG], 1M [ChCl] in EG, and [BMIm][Cl]:EG and, were shown to exhibit substantially lower viscosities than expected when compared to unmodified ILs [17,45]. Figure 5 compares the viscosities of the ILs and DES systems employed for either electroreduction or electro-organic transformation utilising CO_2_.

The selected anion will have a known influence on the viscosity of the ionic liquid as well as the transport of the species to the electrode surface. To demonstrate the influence of anion, selected data on viscosity of the systems that have been commercialized, developed, are gathered and plotted as shown in Figure 5. In a specific case where the anion is paired with the same cation: [BMIm]^+^, the effect of the anion on viscosity follows the trend: [DCA]^−^ < [Br]^−^ < [Tf_2_N]^−^ < [TfO]^−^ < [BF_4_]^−^ < [PF_6_]^−^ < [Ac]^−^. Certain trends from this data may be extrapolated, that hold true regardless of cation’s identity. For instance, results from Fomin et al. [21], Crosthwaite et al. [79] demonstrated that increasing the cationic chain length with common anion [Tf_2_N]^−^ increases the viscosity up to 9000 mPa s. Amongst the literature reported so far, indicated that imidazolium-based ILs are the most studied ILs for electroreduction of CO_2_ electrosynthesis of organic molecules using CO_2_ as reaction media due to their high CO_2_ capture ability, Lewis acid-base interaction [10,14,19,22,36], but the hygroscopic nature of imidazolium cations combined with their high viscosities not only decreases the mass transportation of the reactant species but affect the reduction, electrosynthesis. The majority of studies involving the direct electrochemical conversion of CO_2_ to dialkyl carbonates (such as DMCs) are conducted in imidazolium-based ionic liquids (ILs): [BMIm][BF_4_] and [EMIm][BF_4_] [44]. These solvents are demonstrated to be promising because of the high CO_2_ solubility compared to conventional solvents [0.14, 0.10 vs 0.09 (acetonitrile), 0.07 (ethanol)] at 300 K and 10 bar [51].

Despite these promising yields for both the processes, these systems utilise carcinogenic substances such as [BF_4_]^−^, [PF_6_]^−^ for electroreduction whereas alkylating agents such as methyl iodide and other harmful compounds such as propylene oxide are used for the electrosynthesis of organic carbonates. Subsequently significant efforts were made to eliminate the use of such harmful compounds and reduce the number of production steps. Compton et al. [22] explored the electroreduction of CO_2_ in [BMIm][Ac] which exhibited a high CO_2_ solubility of 1520 mM. The CO_2_ in [BMIm][Ac] underwent a chemically irreversible, one-electron transfer to the radical anion (CO_2_•^−^), and probably favor the formation of oxalate, CO, and carbonate. Yuan et al. [16,57,80] made a series of attempts to substitute the harmful methyl iodide with a variety of basic compounds: CH_3_ONa, NaOH, CH_3_OK, K_2_CO_3_, KOH, to electrosynthesise DMC via CO_2_ transformation on Pt electrodes in dialkyl imidazolium ILs. The results demonstrated that dialkyl imidazolium ILs – (CH_3_OK)–(CH_3_OH) system displayed the highest yield. The authors observed that the higher K^+^ ion interaction with adsorbed CO_2_ resulted in better stabilisation of the CO_2_^−^ anion which favoured the electrosynthesis pathway for the formation of dimethyl carbonates. While carcinogenic chemicals can be avoided, the use of less hazardous ILs has certain limitation such as high cost and viscosity besides low cost. For instance, the viscosity of [BMIm][Ac] is very high (413 mPa) in the case of CO_2_ electroreduction, while the DMC yield achieved with CH_3_OK as alkylating agent was ~4%. Hence, a more cost-efficient and feasible system for the electrochemical reduction of CO_2_ and electrochemical conversions involving CO_2_ to respective carbonates, carbamates, carboxylates and organic compounds needs to be developed.

Evaluating the electrochemical conversion of CO_2_ at higher temperatures in the range of 100 °C employing newly designed ILs, DES systems can result in industrially relevant rates of CO_2_ conversions to ethanol and ethylene besides CO, HCOOH. Such a temperature is expected to decrease the viscosity of the ILs while enhancing the mobility of the reactant species with increased solubility. As a result, high Faradaic efficiencies and current densities can be reproducibly achieved. With key advances in catalysts (2D structures, heteroatom doped structures, single faceted crystal structures) that lead to impressive performance at the lab scale, additional work on these catalysts in newly developed less hazardous non-aqueous systems is required to provide benchmarks against which industries can compare their results. Because ILs are widely acknowledged as one of the most expensive compounds, their cycle stability in the electrochemical reduction process should be taken into account. In addition, the best experimental findings should be obtained at the lowest feasible cost. [EMIm][BF_4_] performed better in electroreduction of CO_2_ to CO and other value-added compounds in several imidazolium-based ILs. However, potential issues arise when [EMIm][BF_4_] is hydrolyzed, which releases HF and certain anions): [BF_3_OH]^−^, [BF_2_(OH)_2_]^−^, [BF(OH)_3_]^−^,and [B(OH)_4_]^−^. This was shown to occur upon addition of water (which is introduced to compensate for viscosity) [36]. The formation of HF makes the CO_2_ saturated solution more acidic and aggressive, corroding the equipment and electrodes, whilst the other complexes increase the reaction rate. Also, hydrolysis of [BMIm][BF_4_] makes the recycling difficult owing to its reduced stability and as a consequence, increases the experimental cost.

To solve this problem, [BF_4_]^−^ anions are replaced with [TfO]^−^ and [BMIm]^+^ as the cation as an alternative [81]. While the imidazolium cations in ILs play a key role in the electrochemical conversion of CO_2_ (reduction, transformation), the anions have an impact on the pricing of imidazolium-based ILs. Currently, there are numerous imidazolium based ILs that exist commercially with different properties and variable costs. Also, there were new developments in DESs as an electrolyte or reaction media for the electrochemical conversion of CO_2_. The high cost associated with ILs is one of the bottlenecks that hinder their industrial use. Hence, it is critical to evaluate and understand the price and economic feasibility of ILs. Besides, with the growing significant interest in DESs it is worth to compare their prices to understand the economic impacts better.

**Figure 6:**
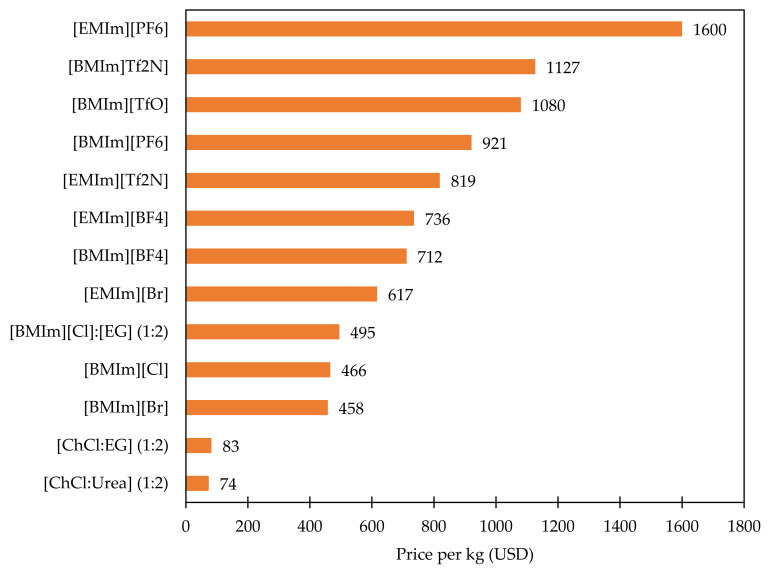
Plot comparing the cost of ILs, DESs in USD per kg based on commercial pricing.

**Figure 7:**
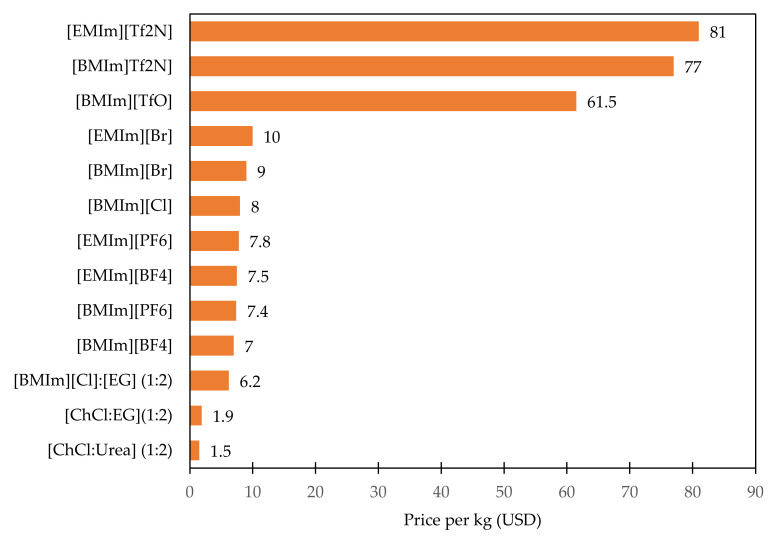
Plot comparing the cost of the ILs, DESs in USD per kg that are computed based on the raw materials cost by adopting the methodology reported by [15].

Figure 6 depicts the plots based on commercially accessible pricing, and Figure 7 is plotted based on the cost of the raw materials utilized in the synthesis of these ILs, DESs and the methodology adopted by Cui et al. [15] in their review. The cost for DESs is evaluated based on their mole ratios of the individual components and the commercially available pricing. Since the price variations per kg of the product are quite significant, the associated costs with ILs, DESs synthesized from raw materials are presented individually. Data from Figure 6 and Figure 7 signify that the cost of ILs paired with [TfO]^−^ or [Tf_2_N]^−^ anion is higher than that of other ILs. These cost comparison data derived from the Figure 6 and Figure 7 validates the fact that the cost of imidazolium-based ILs is mostly determined by the anions, in line with the above analysis. It is worth noting that the price of DESs from Figure 6 and Figure 7 are significantly lower than ILs, which further validates the scientific claims. Also, it might be the primary reason to motivate the industries to focus on their development.

By comparing and evaluating the market pricing of ILs, DESs, it is clear that there exists a significant gap between the price of commercially marketed ILs, DESs plotted in Figure 6 and the price of ILs, DESs produced from raw materials represented in Figure 7. This suggests that increasing the production of ILs, DESs through large scale preparation for the electrochemical conversion of CO_2_ will offer a competitive cost benefit compared with the commercial market pricing, making the possibility to achieve the price of around $1 per kilogram of IL (or) DES [71].

While there are significant efforts in reducing the costs for the conversion processes either by employing co-solvents such as acetonitrile, alcohols or using mixed electrolytes (ILs + cosolvent), the price is still competitive. Therefore, prior attention needs to be paid for their applications in large scale industries. Also, the current commercially available ILs that are employed for the electrochemical conversion of CO_2_ contain carcinogenic, harmful or toxic substances. These substances might need to be replaced with less hazardous ones such as acetate in order for them to comply with the EU regulations and such as Registration, Evaluation, Authorisation and Restriction of Chemicals (REACH).

Besides the predominantly expensive ILs, another commercial challenge is the costs associated with electricity which influences the profitability of the electrochemical processes that utilise CO_2_ either in the electroreduction or electrosynthesis (of organic molecules). Techno-economic model results based on the generalised electrochemical reduction plant developed by Jouny et al. [82] demonstrates that electricity costs influence the net present value significantly. Besides, it also demanded for a continuous supply of cheap electricity to reduce the overall commercial electrochemical CO_2_ reduction plant production costs. One strategy for lowering the production cost would be the use of electricity generation via renewable energy source such as solar. The utilisation of renewable energy source to produce electricity can be considered as an attractive approach to produce carbon feedstocks such as hydrocarbons (formic acid, ethylene) in a carbon-neutral way via the electrochemical routes (reduction, synthesis). Utilising the renewable energy resources such as wind, solar, geothermal energies for the CO_2_ conversion via the electrochemical route can reduce the global weighted-average levelized cost of electricity (LCOE). As per the IRENA reports, solar energy was shown to be the low-cost option that can be used to provide both electrical and thermal energy [83]. Using the solar energy as a source of renewable electricity for the conversion of CO_2_ to produce renewable sources is expected to further reduce the LCOE, production costs and contribute to the carbon neutral economy. Finally, the materials of construction of the suitable equipment used for the electrochemical conversion of CO_2_ needs to be chosen wisely when choosing certain corrosive ILs such as imidazolium-based chlorides. Identifying the alternatives to the commercial hazardous anions and the replacement of imidazolium cations with other family of ILs will be a beneficial approach for rapid commercialization of non-aqueous systems for electrochemical conversion of CO_2_. Similarly, performing the real-time study with the already developed DESs for CO_2_ conversion technologies will provide a detailed insight into the complexities that might be encountered during their commercialization.

While the development of electrochemical conversion of CO_2_ processes seeks significant capital investments, performing the scale-up of electrochemical reactions at a scale spanning between 100–1000 L with the ILs, IL-based mixed electrolytes makes it expensive. Also, the hygroscopic, corrosive nature of the imidazolium chloride-based ILs demands sophisticated equipment to operate and produce them in bulk. There is an urgent need to increase the CO_2_ utilisation to produce products for renewable energy technologies via the electrochemical conversions such as reduction, synthesis (through transformation) while optimising the costs to meet the carbon neutral economy. Furthermore, their compatibility with co-solvents such as water with better recycling characteristics and optimisation of the process intensification technologies will not only reduce the operational cost but also the equipment, overheads.

## 4. Future Perspective

Most of the works covered in this review focused on the combinations of imidazolium, pyrrolidinium cations with different anions: [BF_4_]^−^, [PF_6_]^−^, [TfO]^−^, [Tf_2_N]^−^, [FAP]^−^ with concentrations from pure ILs to millimolar, molar ranges but the optimum compositions were not determined. As these systems pose certain safety hazards such as carcinogenicity, toxicity, bio-degradability, they might have to be replaced with less hazardous category of ILs in order to comply with the safety regulations such as REACH, Restriction of Hazardous Substances (RoHS). Development of halide-free ILs, DESs alternative to imidazolium chlorides (such as acetates), less hazardous alkylating agents (eg.CH_3_OK) can be considered as a way forward.

Few works on the application of halide free IL systems such as acetate-based, and DESs such as [ChCl:Urea/EG], imidazolium chloride-based ones have been investigated as potential alternative electrolytes for the electroreduction of CO_2_ [17]_,_ but studies on the electrosynthesis of organic compounds through the transformation route are still scarce. Also, most of the works focused on the electrosynthesis of carbonates with little on carboxylates, carbamates employing CO_2_-saturated ILs.

Hence, a more cost-effective system to convert CO_2_ to the respective organic compounds such as CO, HCOOH, ethylene (C_2_H_4_), and carbonates, carboxylates, carbamates through electrochemical approach needs to be developed. Identification of the reaction intermediates through mechanistic understanding during the electrochemical conversion of CO_2_ in either ways will be the key challenge [9,15,50]. However: (i) enhancing the real time performance of low-cost halide free ILs (such as acetate), and imidazolium chloride- based DESs, (ii) accelerating the research on ILs’ recovery and reuse will be a significant future challenge.

Preliminary screening of the alternatives based on their properties (physical, chemical, electrochemical), safety, cost from a variety of ILs, DESs could reduce the time and efforts in identifying the best ones. Utilising such electrolytes for either of the electrochemical conversions: CO_2_ reduction, transformation via synthesis will help to increase the performance of CO_2_ utilization technologies besides promoting the scope of ILs/DESs in the renewable energy sector.

## 5. Conclusions

The present review summarised the developments in electrochemical conversion of CO_2_ value added chemicals through the electroreduction and electroorganic transformation using ILs, DESs as the electrolyte. Imidazolium and pyrrolidinium cations have been proved to be very effective for enhancing the performance of the catalysts towards the electroreduction of CO_2_, with imidazolium-based ILs being predominant. While the cations of ILs play a multifunctional role in the electroreduction system, interacting with reaction intermediates and possibly acting as a co-catalyst, the anions contribute to electrolyte stability but increase the overall cost. The studies referenced reflect the scientific community’s significant attempts to produce efficient electrochemical CO_2_ conversions, either through reduction or transformation.

Having understood the progress in the electrochemical conversion of CO_2_ employing ILs, DESs, the techno-commercial challenges, the non-aqueous electrolyte based approach could be considered as a promising way forward for obtaining the resultant products (HCOOH, HCHO, C_2_H_4_ etc.). These products serve as a renewable source to produce value added chemicals at industrial scale. Clearly, significant improvements are desired in terms of identifying the less hazardous ILs, scope of DESs for electrochemical conversion of CO_2_ to make a step towards establishing such technology on a larger scale and demonstrating it as a sustainable process. While substantial improvements have been recorded in understanding the mechanistic aspects of CO_2_ electrochemical transformations in ILs, investigations using DESs as the electrolyte deserve considerable research.

The primary challenges to focus at this stage have been outlined in this review which is expected to potentially support the research community working in this field and aid with rapid commercialisation of ILs/DESs.

## Figures and Tables

**Table 1:** List of ionic liquids (ILs) discussed in this review.

Ionic Liquid	Chemical Formula	Cation	Anion	Abbreviation	Ref.
1–butyl–3–methyl imidazolium bromide	[(C_8_H_15_N_2_Br)]	[(C_4_H_9_)(CH_3_)(C_3_H_3_N_2_)]^+^	[Br]^−^	[BMIm][Br]	[16]
1–butyl–3–methyl imidazolium chloride	[(C_8_H_15_N_2_Cl)]	[(C_4_H_9_)(CH_3_)(C_3_H_3_N_2_)]^+^	[Cl]^−^	[BMIm][Cl]	[17]
1–ethyl–3–methyl imidazolium bis(trifluoro methyl sulfonyl) imide	[(C_8_H_11_F_6_N_3_O_4_S_2_)]	[(C_2_H_5_)(CH_3_)(C_3_N_2_H_4_H)]^+^	[CF_3_SO_2_CF_3_SO_2_N]^−^	[EMIm][Tf_2_N]	[18]
1–propyl–1–methyl imidazolium bis(trifluoro methyl sulfonyl) imide	[(C_9_H_13_F_6_N_3_O_4_S_2_)]	[(C_3_H_7_)(CH_3_)(C_3_N_2_H_4_H)]^+^	[CF_3_SO_2_CF_3_SO_2_N]^−^	[PMIm][Tf_2_N]	[18]
1–butyl–3–methyl imidazolium bis(trifluoro methyl sulfonyl) imide	[(C_10_H_15_F_6_N_3_O_4_S_2_)]	[(C_4_H_9_)(CH_3_)(C_3_N_2_H_4_H)]^+^	[CF_3_SO_2_CF_3_SO_2_N]^−^	[BMIm][Tf_2_N]	[19]
1–butyl–3–methyl imidazolium hexa fluorophosphate	[(C_8_H_15_F_6_N_2_P)]	[(C_4_H_9_)(CH_3_)(C_3_N_2_H_4_H)]^+^	[PF_6_]^−^	[BMIm][PF_6_]	[19]
1–butyl–3–methyl imidazolium tetrafluoroborate	[(C_8_H_15_F_4_N_2_B)]	[(C_4_H_9_)(CH_3_)(C_3_N_2_H_4_H)]^+^	[BF_4_]^−^	[BMIm][BF_4_]	[19]
1–butyl–3–methyl imidazolium trifluoro methane sulfonate	[(C_9_H_15_F_3_N_2_O_3_S)]	[(C_4_H_9_)(CH_3_)(C_3_N_2_H_4_H)]^+^	[CF_3_SO_3_]^−^	[BMIm][TfO]	[19]
1–butyl–3–methylimidazolium tris(perfluoroethyl)trifluorophosphate	C_14_H_15_FN_2_P	[(C_4_H_9_)(CH_3_)(C_3_N_2_H_4_H)]^+^	[(C_2_F_5_)_3_PF_3_]^−^	[BMIm][FAP]	[19]
1–butyl–3–methylimidazolium dicyanamide	[(C_10_H_15_N_5_)]	[(C_4_H_9_)(CH_3_)(C_3_N_2_H_4_H)]^+^	[C_2_N_3_] ^−^	[BMIm][DCA]	[19]
1–butyl–1–methyl pyrrolidine bis(trifluoro methyl sulfonyl) imide	[(C_11_H_20_F_6_N_2_O_4_S_2_)]	[(C_4_H_9_)(CH_3_)C_4_H_9_NH)]^+^	[CF_3_SO_2_CF_3_SO_2_N]^−^	[BMPyl][Tf_2_N]	[20]
1–ethyl–3–methyl imidazolium hexa fluorophosphate	[(C_6_H_11_F_6_N_2_P)]	[(C_2_H_5_)(CH_3_)(C_3_N_2_H_4_H)]^+^	[PF_6_]^−^	[EMIm][PF_6_]	[21]
1–ethyl–3–methyl imidazolium tetrafluoroborate	[(C_6_H_11_F_4_N_2_B)]	[(C_2_H_5_)(CH_3_)(C_3_N_2_H_4_H)]^+^	[BF_4_]^−^	[EMIm][BF_4_]	[21]
1–ethyl–3–methyl imidazolium trifluoro methane sulfonate	[(C_7_H_11_F_3_N_2_O_3_S)]	[(C_2_H_5_)(CH_3_)(C_3_N_2_H_4_H)]^+^	[CF_3_SO_3_]^−^	[EMIm][TfO]	[21]
1–butyl–3–methyl imidazolium acetate	[(C_10_H_18_N_2_O_2_)]	[(C_4_H_9_)(CH_3_)(C_3_N_2_H_4_H)]^+^	[CH_3_COO]-	[BMIm][Ac]	[22]

**Table 2:** Summary of IL -based electrolytes used in the electroreduction of CO_2_.

Electrolyte	Catalyst	Reactor Type	Major Products	Faradaic Efficiency (FE, %) ^1^	Reference
[BMIm]BF_4_	N-doped carbon (graphene-like) materials/carbon paper electrodes	H-Cell	CH_4_	93.50	[19]
[BMIm][Ac]	Platinum	Two electrode cell	Oxalate, CO, carbonate	-	[22]
18 mol % [EMIm][BF_4_] in H_2_O	Silver nanoparticles	Flow cell	CO, H_2_	96	[28]
[BMim][PF_6_]	Copper plank	High pressure undivided cell	CO, H_2_, HCOOH (traces)	90.20	[29]
4 mol % [EMIm][BF_4_] + 96 mol % H_2_O	Molybdenum disulphide	Custom made 2 compartment three electrode cell	CO	98	[31]
[EMIm][BF_4_]/H_2_O (50 vol %/50 vol %)	WSe_2_ nanoflakes	2 compartment 3- electrode electrochemical cell	C/O	24	[32]
25 mol % [EMIm][BF_4_] + H_2_O (75 mol %)	Metal free carbon nanofibers	3-electrode electrochemical cell	CO	98	[33]
[EMIm][BF_4_]: H_2_O (1:1 *v*/*v*)	Nanostructured and nanosized Titania	H-cell	Low density poly ethylene (LDPE)	14	[35]
10.5 mol % [EMIm][BF_4_] + 89.5 mol % H_2_O	Silver nanoparticles	Flow cell	CO	100	[36]
[EMIm][BF_4_]	Silver nanoparticles	Flow cell	CO	-	[37]
[BMIm][BF_4_]	Flat platinum spirals	2-compartment homemade glass cell	NHC ^2^–CO_2_ adduct	-	[38]
80 wt % [BMIm][Cl] + 20 wt % H_2_O	Silver	H-Cell	CO	>99	[39]
[BMIm][BF_4_]	Indium tin oxide	Undivided glass electrochemical cell	CO	64.90	[40]
[EMIm][Tf_2_N}	Pre-anodized Pt electrode	Two electrode cell	HCOOH	-	[41]
[BMPyr][Tf_2_N}	Pre-anodized Pt electrode	Two electrode cell	HCOOH	-	[41]
[EMIm][BF_4_]/H_2_O (92/8 *v*/*v* %)	Silver nanoflowers	Flow cell	CO	75	[42]

^1^ The values are indicated based on the optimized conditions reported by the referenced works. ^2^ NHC: N-heterocyclic carbene.

**Table 3:** Summary of DESs used in the electroreduction of CO_2_.

Electrolyte	Catalyst	Reactor Type	Major Products	Faradaic Efficiency (FE, %)	Reference
[ChCl]^1^[Urea] (1:2)	Silver	U-type divided cell	CO	15.80	[17]
[ChCl][Urea] (1:2) + H_2_O (15 vol %)	Silver	U-type divided cell	CO	59	[17]
[ChCl]–EG ^1^ (1:2)	Silver	U-type divided cell	CO	78	[17]
[BMIm][Cl]:[EG] (1:2)	Silver	U-type divided cell	CO	95.80	[17]
1M [ChCl] in EG	Silver	U-type divided cell	CO	71.10	[17]
1M [ChCl] in PEG-200	Silver	U-type divided cell	CO	83.20	[17]
1M [BMIm][Cl] in PEG-200	Silver	U-type divided cell	CO	85.90	[17]
2M [ChCl]{Urea] (1:2)	Silver	Electrochemical flow reactor	CO	94.10	[45]
[ChCl][Urea] (1:2) + H_2_O (50 vol %)	Silver	H-cell	CO	96	[47]
[MEAHCl][MDEA] ^2^	Silver	Three electrode cell	CO	71	[48]

^1^ ChCl: choline chloride; EG: ethylene glycol. ^2^ [MEAHCl][MDEA]: [monoethanolamine hydrochloride] [methyl diethanolamine].

**Table 4:** Summary of IL-based electrolytes used in the electrochemical conversion of CO_2_ via the transformation route.

Product Family	Product	Electrolyte	Substrates	Cathode	Anode	Reactor Type	Reference
Dialkyl carbonate	Dimethyl carbonate	[BMIm][Br]	Methanol and propylene oxide	Platinum	Platinum	One compartment cell	[16]
Carboxylate	2–hydroxy–2–phenyl propionic acid	[BMM’Im] [BF_4_]	Acetophenone	Glassy carbon (cylindrical tube)	Magnesium	Undivided cell	[20]
	2–hydroxy–2–phenyl propionic acid	[BMPy] [Tf_2_N]	Acetophenone	Glassy carbon (cylindrical tube)	Magnesium	Undivided cell	
Cyclic carbonate	Styrene carbonate	[BMIm][BF_4_]	Styrene, glycol, methyl iodide (alkylating agent), potassium carbonate	Titanium	Platinum spiral	Two compartment cell divided by a cation exchange membrane	[55]
Dialkyl carbonate	Dimethyl carbonate	[B’MIm][Cl]	Methanol	Graphite	Platinum	Undivided cell (four neck bottle)	[57]
Carboxylate	Phenyl acetic acid	[BMIm][BF_4_]	Benzyl chloride	Silver cylinder	Magnesium	Undivided cell	[58]
Carboxylate	2–phenyl propionic acid	[DEME] [Tf_2_N]	α–chloroethyl benzene	Platinum plate	Magnesium	High pressure vessel	[59]
Dialkyl carbonate	Dimethyl carbonate	[BMIm][BF_4_]	Methanol; methyl iodide (alkylating agent)	Silver-coated nanoporous copper	Platinum foil	Undivided cell	[60]
Carbamate	6–amino nicotinic acid	[BMIm][BF_4_]	2–amino–5– bromopyridine	Silver	Magnesium rod	Undivided cell	[61]
Carbamate	Organic carbamates	[BMIm][BF_4_]	Amines, O_2_, ethyl iodide (alkylating agent)	Copper	Platinum spiral	Two compartment 3-electrode cell	[62]
Dialkyl carbonate	Dimethyl carbonate	[AMIm][Br]	Methanol	Graphite	Platinum	Undivided cell (four neck bottle)	[63]
Dialkyl carbonate	Dimethyl carbonate	[BMIm][Br]	Potassium ethoxide	Platinum/ niobium plates	-	Divided electrochemical cell	[64]
Dialkyl carbonate	Dimethyl carbonate	[BMIm][Br]	Potassium ethoxide	Graphite	-	Divided electrochemical cell	[65]

## Data Availability

The data presented in this study are available upon request from the corresponding author.
